# Estrogen regulation of cardiac cAMP-L-type Ca^2+^ channel pathway modulates sex differences in basal contraction and responses to β_2_AR-mediated stress in left ventricular apical myocytes

**DOI:** 10.1186/s12964-019-0346-2

**Published:** 2019-04-15

**Authors:** Jeremiah Ong’achwa Machuki, Hong-Yuan Zhang, Juan Geng, Lu Fu, Gabriel Komla Adzika, Lijuan Wu, Wenkang Shang, Jinxia Wu, Li Kexue, Zhiwei Zhao, Hong Sun

**Affiliations:** 1 Physiology Department, Xuzhou Medical University, Xuzhou, 221004 Jiangsu China; 2 Institute of Cardiovascular Disease Research, Xuzhou Medical University, Xuzhou, 221002 China

**Keywords:** Ca^2+^ transients, Cardiac stress, 17β-estradiol, L-type Ca^2+^ channel, Phosphodiesterase, Sex differences in contractility

## Abstract

**Backgrounds/Aim:**

Male and female hearts have many structural and functional differences. Here, we investigated the role of estrogen (E2) in the mechanisms of sex differences in contraction through the cAMP-L-type Ca^2+^channel pathway in adult mice left ventricular (LV) apical myocytes at basal and stress state.

**Methods:**

Isolated LV apical myocytes from male, female (Sham) and ovariectomised mice (OVX) were used to investigate contractility, Ca^2+^ transients and L-type Ca^2+^ channel (LTCC) function. The levels of β_2_AR, intracellular cAMP, phosphodiesterase (PDE 3 and PDE 4), RyR2, PLB, SLN, and SERCA2a were compared among the experimental groups.

**Results:**

We found that (1) intracellular cAMP, *I*_*CaL*_ density, contraction and Ca^2+^ transient amplitudes were larger in Sham and OVX + E2 myocytes compared to male and OVX. (2) The *mRNA* expression of PDE 3 and 4 were lower in Sham and OVX + E2 groups compared with male and OVX groups. Treatment of myocytes with IBMX (100 μM) increased contraction and Ca^2+^ transient amplitude in both sexes and canceled differences between them. (3) β_2_AR-mediated stress decreased cAMP concentration and peak contraction and Ca^2+^ transient amplitude only in male and OVX groups but not in Sham or OVX + E2 groups suggesting a cardioprotective role of E2 in female mice. (4) Pretreatment of OVX myocytes with GPR30 antagonist G15 (100 nM) abolished the effects of E2, but ERα and ERβ antagonist ICI 182,780 (1 μM) did not. Moreover, activation of GPR30 with G1 (100 nM) replicated the effects of E2 on cAMP, contraction and Ca^2+^ transient amplitudes suggesting that the acute effects of E2 were mediated by GPR30 via non-genomic signaling. (5) *mRNA* expression of RyR2 was higher in myocytes from Sham than those of male while PLB and SLN were higher in male than Sham but no sex differences were observed in the *mRNA* of SERCA2a.

**Conclusion:**

Collectively, these results demonstrate that E2 modulates the expression of genes related to the cAMP-LTCC pathway and contributes to sex differences in cardiac contraction and responses to stress. We also show that estrogen confers cardioprotection against cardiac stress by non-genomic acute signaling via GPR30.

## Background

Male and female hearts have well-known structural and functional differences [1–3]. However, investigations on the mechanisms that underlie these differences have not yielded a consistent conclusion. Differences in β-adrenergic receptor (βAR) responses [4], L-type Ca^2+^ channel (LTCC) function, sarcoplasmic reticulum (SR) Ca^2+^ release dynamics [5], and age [6] have been shown to contribute to sex differences in many animal models. Yet, the cause of these variations remains unsettled, as such, and uncovering them is the focus of this study. It is emerging from prior studies that sex differences in cardiac contraction may be species-specific and/or dependent on multilevel effects of sex hormones [7]. The variation in plasma 17β-estradiol (E2) is known to alter cardiac output [8, 9]. In our previous study, we found that E2 influences expression and signaling cascades of β_1_AR and β_2_AR, the two main regulators of cardiac contraction [10, 11]. Therefore, we speculate that E2 may be modulating signaling cascades of the cardiac contraction system. This may explain the sex differences observed in cardiac contraction [7].

βARs transmit signals to various Ca^2+^-handling proteins mainly through the Gs/Gi -cAMP-PKA pathways [12]. cAMP-activate PKA, which phosphorylates LTCC leading to entry of Ca^2+^ (*I*_*CaL*_) which triggers SR Ca^2+^ release through RyR2. The resultant rise in cytosolic Ca^2+^ (Ca^2+^ transient) activates myofilaments leading to contraction [13]. For relaxation to occur, Ca^2+^ is pumped back to SR by SERCA2a which is regulated by Sarcolipin (SLN) and phospholamban (PLB). Hence, intracellular cAMP level is an important determinant of the activity of LTCCs, RyR2, SERCA2a, SLN, and PLB [12]. Despite the fact that cAMP signals are elicited by both β_1_ARs and β_2_ARs, it is known that β_1_AR is more efficient at increasing cardiac contractility than β_2_AR [14]. Several post-receptor factors affect the progression of βARs-induced signaling to ensure cardiac contraction occurs. One of such factors is the balance between synthesis of cAMP and its breakdown by cyclic-nucleotide phosphodiesterases (PDEs) [15]. PDE2–4 play major roles in cAMP compartmentation in adult cardiomyocytes [16]. Indeed, PDE2 and PDE3 are key determinants of basal cardiac contraction while PDE4 predominate cAMP regulation during β-adrenergic stimulation [15]. Hence, investigating whether there are sex differences in the cardiac expression of PDEs will help to elucidate if it plays a role in contractile differences between sexes.

The role of E2 (through its receptors; ERα, ERβ, and GPR30) and the modulation of β_2_AR in the heart are integral to the mechanisms that orchestrate cardioprotection. In the apex of the left ventricle, β_2_AR outnumbers β_1_AR whereas β_1_AR outnumbers β_2_AR at the base [17]. β_2_AR mediates stress induced by high catecholamine which produces hypocontractility from the mid left ventricle to the apex in Takotsubo cardiomyopathy. E2 on the other hand protects against stress-induced cardiomyopathy [18, 19]. Indeed, the loss of E2 in postmenopausal women is thought to account for the high prevalence of cardiovascular diseases in this group compared to men and premenopausal women [20]. But, the role of E2 in sex differences in cardiac contraction is not clear. In this study, we examined the effects of E2 on β_2_AR, cAMP, LTCC, PLB, SERCA2a, RyR2, SLN, PDE3, and PDE4 families) using apical myocytes from adult male and female left ventricle (LV). Cardiac stress was induced by high epinephrine treatment. In identifying the specific E2 receptor (s) mediating the roles of E2, G1 (agonist) and ER antagonists (G15 and ICI 182,780) were used.

## Materials and methods

### Animals

Sexually mature, weight-matched wild-type and β_2_AR-knock out FVB mice (kindly donated by Professor Daniel Bernstein in (Stanford University, USA)) were used for the experiments. All animals were housed in standard cages at a temperature of 23 ± 2 °C and a controlled environment with 12-h/12-h dark-light cycle. Mice were fed on pelleted food and water ad libitum. Adult cardiomyocytes have an advantage over neonatal cardiomyocytes given that stress cardiomyopathy and heart failure occur almost exclusively in the adult population.

### Materials

17β-estradiol (E2), epinephrine (Epi), isoproterenol (ISO), and non-selective PDE inhibitor (IBMX) were purchased from Sigma Aldrich (St. Louis, MO, USA). Fura-2 AM was purchased from WesTang Bio-technology (Shanghai, China). Alkaline phosphatase Goat anti-Rabbit IgG was purchased from ZSGB-BIO (China). GAPDH Polyclonal Rabbit anti-mouse antibody was obtained from ABclonal (Wuhan, China). The anti-Cav1.2α was purchased from (L-type Ca^2+^ channel subunit a1c; Alomone Lab, Israel). ERα and ERβ antagonist ICI 182,780 (ICI) (Sigma), GPR30 agonist (G1) and antagonist (G15) were purchased from Cayman chemical (USA) and were solubilized in DMSO.

### Isolation of mice left ventricular apical myocytes

Animal body weight and wet heart weight were measured prior to myocyte isolation. Adult LV apical cardiomyocytes were dissociated using Collagenase B and D (Roche Diagnostics GmbH, Mannheim, Germany) and Protease type XIV (Sigma) as described by Zhou et al. [21], and it was optimized as follows (1) Animals were heparinized and anesthetized using Chloral hydrate and the entire digestion process was performed on the largendorff apparatus, (2) 20 mM taurine was used in all buffer solutions, (3) The hearts were submerged in ice-cold Ca^2+^-free buffer immediately after excision, (4) Another step was added during Ca^2+^ re-introduction phase with 0.75 mM Ca^2+^ to allow myocytes to acclimatize. (5), Myocytes from the apex of LV were taken from 3 to 4 mm from the very bottom of the heart. The myocytes were stored in a HEPES-buffered solution consisting of (in mM) 1 CaCl_2_, 137 NaCl, 5.4 KCl, 15 Glucose, 1.3 MgSO4, 1.2 NaH_2_PO4, and 20 HEPES, adjusted to pH 7.4 with NaOH at room temperature. Only elongated myocytes with clear striations and quiescent when unpaced were considered healthy for experiments.

### Determination of plasma estrogen levels

Adult ovulating female mice were selected by examination of the vaginal opening and vaginal cell typology as surrogate indicators of the underlying changes of the estrous cycle as described previously [22]. Blood was collected after excision of the heart at the time of cardiomyocytes isolation, and serum was stored at − 80 °C until the day of assay. Plasma E2 was determined by radiolabeling assay kit (Jiuding Biological Engineering Company, Tianjin, China) according to the manufacturer’s instructions.

### Mice stress model (β_2_AR-mediated)

A pilot study was performed to determine contractile response at the indicated concentrations of isoproterenol (ISO) (10^− 4^, 10^− 5^, 10^− 6^, 10^− 7^, 10^− 8^ M) in male left ventricular myocytes. Briefly, few drops of medium containing myocytes were added to the superfusion chamber on the stage of an inverted microscope (Olympus, Tokyo, Japan). The cardiomyocytes were allowed to settle to the bottom of the chamber after which they were continuously superfused with a HEPES-buffered solution consisting of (in mM) 1 CaCl_2_, 137 NaCl, 5.4 KCl, 15 Glucose, 1.3 MgSO4, 1.2 NaH_2_PO4, and 20 HEPES at 23 °C. Myocytes were stimulated at 0.5 Hz via platinum field electrodes in the absence/presence of ISO in HEPES-buffered solution. The contraction amplitude at each concentration of ISO was normalized to the basal amplitude. Peak amplitude shortening was recorded at 1 μM of ISO and hence this concentration was chosen for βAR stimulation throughout the study. Stress was simulated by pre-stimulation of β_2_AR with high epinephrine (Epi). Briefly, myocytes were pre-incubated with 1 μM Epi in HEPES-buffered solution at 23 °C. At the duration of 10, 20, 40, and 50 min, their response to ISO stimulation was determined as previously described by Paur et al. [23]. β_2_AR-specific effects were confirmed in β_2_AR-gene knockout (β_2_KO) cardiomyocytes.

### Determination of myocyte shortening and Ca^2+^ transients

Measurement of cardiomyocyte contraction and Ca^2+^ transients was performed as described before [24]. Briefly, cardiomyocytes were incubated with 1 μM acetoxymethyl ester of Fura-2 (Beyotime Biotechnology, Beijing, China) contained in the HEPES-buffered solution in a dark room at 23 ± 1 °C for 25 min and thereafter the cells were washed three times with a fresh HEPES buffer (23 °C, pH 7.4) before experimentation. To investigate the effects of 1 nM E2, G1 (100 nM), G15 (100 nM), ICI 182,780 (1 μM), on contraction and Ca^2+^ transients, myocytes were co-incubated with these drugs in combinations shown in figures for 1 h at room temperature. For IBMX treatment, myocytes were incubated with IBMX (100 μM in DMSO) for 10 min at room temperature prior to experimentation. Cytosolic Ca^2+^ transient (Fura-2 fluorescence) and cell length changes were recorded simultaneously using the IonOptix software (IonOptix Corp., Milton, MA, USA) with 0.5 Hz electrical stimulation at 23 °C. Signals from the video edge system were digitized and stored in a computer. At the start of each recording, resting cell length was measured with the edge detector to allow normalization of contraction during analysis. All data were recorded 30 s after stimulation to ensure only stable data were captured. Only rod-shaped cells, without blebs or other visible structural alterations, which responded regularly to stimulation were used for this experiment. At least 10 regularly contracting myocytes were recorded for each group and contraction was presented as fractional shortening normalized to cell length and Ca^2+^ transient amplitudes were calculated as the percentage difference between peak amplitude and baseline amplitude. Fluorescence data were analyzed using the IonOptix acquisition software. For determination of myocyte shortening of Fura-2 AM unloaded myocytes, cells were added to the stage of an inverted microscope (Olympus, Tokyo, Japan) and stimulated with 0.5 Hz electrical stimulation at 23 °C. Data analysis was performed using Optical Measure software (kindly provided by China’s National Defense University of Science and Technology).

### Generation of OVX mice and measurement of body and heart weight

Ovariectomy is often used to induce artificial menopause. Weight-matched wild-type and β_2_AR-deficient female mice were randomly assigned to Sham and OVX groups. Mice were anesthetized with Chloral hydrate and ovariectomized after a single midline dorsal skin incision, 3 cm long (OVX group), but for the Sham group, mice were subjected to similar treatments except for the removal of ovaries. Mice were allowed 2**–**3 weeks to recover from the surgery. The body and wet heart weights were measured at the time of cardiomyocytes isolation (Table 1).

**Table 1:** The physical characteristics of male and female mice

Attributes	Wild-type Mice		β_2_AR-knockout Mice	
	Male	Female	Male	Female
Body weight, (g)	25.2 ± 0.66	25 ± 1.04	26.8 ± 1.11	29.2 ± 1.31
Heart wet weight, (g)	0.1499 ± 0.01	0.1303 ± 0.01	0.1453 ± 0.00	0.1495 ± 0.01
Cell capacitance	Male	Female	OVX	
	163.4 ± 6.70	147.2 ± 13.10	180.0 ± 12.80	

Body weight and heart weight of male and female wild-type and β_2_AR-knockout mice. There were no significant differences in body weight and heart wet weights between male and female mice. The data are presented as means ± SEM

### Patch clamp experiments

Single, Ca^2+^ tolerant and rod-shaped myocytes were suitable for whole-cell *I*_*CaL*_ measurements. Voltage-clamp mode was used in the recording of membrane currents. *I*_*CaL*_ recording was performed using PatchMaster (v2x69) software (HEKA Instruments) and EPC10 amplifier. Pipette had tip resistance of 3–5 mΩ when filled with a solution containing the following (in mM) 120 CsCl, 10 EGTA, 5 Na_2_ATP, 10 HEPES (pH 7.2 by CsOH) and was used to form a high-resistance seal to myocyte membrane. A negative pressure was applied to form a whole-cell configuration by rupturing the membrane. *I*_*CaL*_ was recorded with an appropriate stimulus protocol. The external bath solution for recording *I*_*CaL*_ was (in mM) 133.5 NaCl, 4 CsCl, 1.2 MgCl_2_, 11.1 Glucose, 10 HEPES, 1.8 CaCl_2_ (pH 7.4 by NaOH, at 37 °C). *I*_*CaL*_ currents were filtered at 2 kHz and sampled at 50 kHz using PatchMaster (v2x69) software (HEKA Instruments) and recorded using an EPC10 amplifier. The *I*_*CaL*_ was then evoked by voltage pulses of 250 ms between − 40 and + 45 mV in 5 mV increments after inactivation of the I_Na_ following 100-ms voltage steps from the holding potential of − 100 to − 40 mV. All recordings were made immediately after gaining whole cell access to avoid the run-down effect of *I*_*CaL*_. Capacitance measurements were obtained from membrane test parameters. The capacitance of adult male, female, and OVX myocyte capacitance was 163.4 ± 6.7, 147.2 ± 13.1, and 180.0 ± 12.8, respectively. *I*_*CaL*_ were normalized to cell capacitance (pA/ pF). Series resistance was compensated 60% to prevent large voltage errors.

### Western blot for LTCC (Cav1.2α) protein expression

Whole-cell cardiomyocytes lysate were prepared from the following groups male, female, OVX, and OVX + E2 (incubated with E2 after myocytes isolation for 5 h) using 1 ml of RIPA buffer (NP-40, 1%; Na-deoxycholate, 12 mM; SDS, 0.1%; in Phosphate Buffer Saline) for 2 × 10^6^ cells plus 2× protease inhibitor cocktail (Sigma). Protein sample concentrations were determined by the Bicinchoninic Acid (BCA) Assay Kit (Beyotime Biotechnology, Beijing, China). The samples were diluted in loading buffer (130 mM Tris–HCl, pH 8.0, 20% glycerol, 5% sodium dodecyl sulfate (SDS), 0.02% bromophenol blue, 2% DTT) and denatured for 10 min at 100 °C. Equal samples (20 μg) were separated by 10% polyacrylamide gel electrophoresis in the presence of SDS and transferred to nitrocellulose membranes. Non-specific binding was blocked with 4% non-fat milk and the membranes were immunoblotted overnight at 4 °C with primary antibodies against the following Cav1.2α (Lot. ACC003AN5502, 1:200, Alomone Labs), Israel), and β-actin (1:1000, TA-09, Zhongshan, Beijing, China). Subsequently, the membrane was incubated with corresponding secondary antibodies (Alkaline Phosphatase Goat anti-Rabbit IgG (H + L),1:4000, ZB-2308, Zhongshan, Beijing, China) at room temperature for 2 h. Protein bands were visualized using nitro blue tetrazolium and 5-bromo-4-chloro-3-indolyl-phosphate. The membranes were scanned, and the relative intensity of the immunoblots was analyzed by software Photoshop (Adobe, San Jose, CA, USA). A blank area on the nitrocellulose membrane was scanned to subtract the background. Mouse β-actin was used as a positive control for protein presence.

### cAMP enzyme immunoassay

The level of cAMP levels was determined as described before using ELISA kit (Shanghai Jiang Lai Biological Technology Co., Ltd.). Myocytes from each group were incubated in 96-well plates (1000 cells/well) and with 1 nM E2, G1 (100 nM), G15 (100 nM), and ICI 182,780 (1 μM) in combinations shown in the figures for 1 h in the presence/absence of 1 μM ISO, 1 μM Epi for 40 min in HEPES buffer at room temperature. Thereafter, they were centrifuged to form a pellet (4000 rpm for 10 min) and resuspended in PBS and kept at − 80 °C for 14 days. Myocytes cellular membrane rupture was achieved by a freeze-thaw method and intracellular cAMP levels were determined using an ELISA kit. Absorbance was measured at 450 nm by an ELISA plate reader and cAMP levels were calculated from a standard curve (r^2^ = 0.96) and normalized to the quantity of protein in each sample.

### Quantitative real-time PCR- RNA isolation and cDNA synthesis

Isolated male and female myocytes were used to isolate RNA using the TRIzol® reagent (Invitrogen Co., Carlsbad, CA, USA). The purity and concentration of total RNA (200 ng) were measured using NanoDrop 1000 (Thermo Scientific). Freshly isolated OVX myocytes were incubated with 1 nM E2 after 1 h of acclimatization for 5 h and before RNA isolation. cDNA was synthesized using ReverTra Ace qPCR RT kit (Vazyme Biotech, Nanjing, China) by reverse transcription of total RNA. The intron-spanning primers for all genes were synthesized by Sangon Biotech (Shanghai, China) Co., Ltd. All primers are shown in Table 1. Analysis of the cDNA was done in duplicate using 0.2 μmol/L specific primers (Table 2) and 1× LightCycler 480 SYBR green I Master (Roche Applied Science, Germany) in a total volume of 10 μl. GAPDH was used as reference gene. PCR was performed using a Light Cycle 480 (Roche Applied Science, Germany) using the following thermal procedure 5 min at 95 °C followed by 40 cycles of 10 s at 95 °C, 30 s at 60 °C, a melting curve of 15 s at 95 °C, 60 s at 60 °C and cooling for 30 s at 4 °C. Analysis of the results was performed using the LightCycler 480 software (version 1.5, Roche Applied Science, Germany). The relative levels of *mRNA* were analyzed using the 2^ΔΔCt^ method. Quantitative *mRNA* level of all genes was normalized to the housekeeping gene GAPDH which were similar in all groups.

**Table 2:** Primer sequences for quantitative RT PCR

Gene name		Primer sequence (5′ → 3′)		Amplicon length [bp]
		Forward	Reverse	
Pde3a	NC_000072.6 Reference GRCm38.p4 C57BL/6J	TTTAACGCCAAGGTAAACGATG	TGATGTCAGCCAGCTTTATACA	97
Pde3b	NC_000073.6 Reference GRCm38.p4 C57BL/6J	GAAAAAGTGCCTGTGATCAGAC	TCTGTTCTCGGGAAATACAAGG	130
Pde4a	NC_000075.6 Reference GRCm38.p4 C57BL/6J	GCTGACCTGAAGACTATGGTAG	GATACGGTCAGAGTAGTTGTCC	81
Pde4b	NC_000070.6 Reference GRCm38.p4 C57BL/6J	CAGGAAAATGGTGATTGACATGGTGTTGG	CGAAGAACCTGTATCCGGTCAGTATAG	
Pde4d	NC_000079.6 Reference GRCm38.p4 C57BL/6J	CCAGAATCTGACCAAAAAGCAA	GATCCTGTCGGAGTAGTTATCC	175
Atp2a2	NC_000071.6 Reference GRCm38.p4 C57BL/6J	TTCTGCTTATCTTGGTAGCCAA	CTTTCTGTCCTGTCGATACACT	125
Sln	NC_000075.6 Reference GRCm38.p4 C57BL/6J	ATGGAGAGGTCTACTCAGGAGCTG	ACCTCACGAGGAGCCACATAAGG	82
Adrb2	NC_000084.6 Reference GRCm38.p4 C57BL/6J	CACAAAGCCCTCAAGACTTTAG	CCTGATAACGTGCACGATATTG	93
Ryr2	NC_000079.6 Reference GRCm38.p4 C57BL/6J	CCATTGAGAACAAGTACATGCC	AATTCGTTGTTCATCATGAGCC	112
Plb	NC_000076.6 Reference GRCm38.p4 C57BL/6J	TACCTCACTCGCTCGGCTATCAG	CACAATGATGCAGATCAGCAGCAG	132
Cacna1c	NC_000072.6 Reference GRCm38.p4 C57BL/6J	GAGACAATGTGTGGAATATGCC	GTAGGTAGAGTTGACCACGTAC	103

### Statistical analysis

The value n denotes the number of mice/cells used in each experiment. All data analyses were represented as means ± SEM. Statistical analysis and construction of figures were performed with GraphPad Prism 5.01 (San Diego, CA, USA). Statistical significance was estimated by one-way ANOVA followed by Student’s t-test to determine group differences. Spearman rank correlation test was performed to determine the relationship between plasma E2 and contraction amplitude shortening. Differences between groups were considered significant for *P <* 0.05.

## Results

### E2 modulates sex differences in contraction and Ca^2+^ transient amplitude in LV apical myocytes

**Fig. 1:**
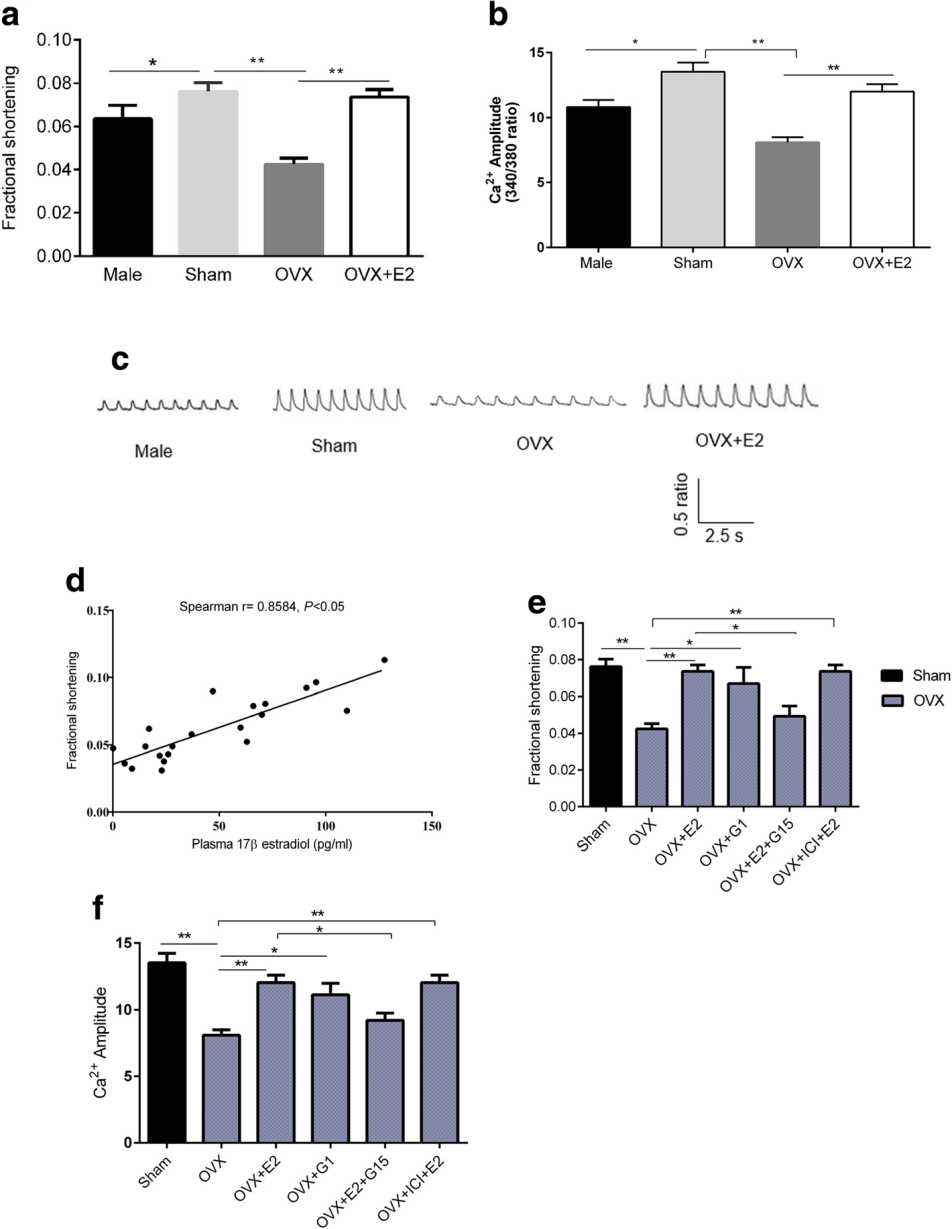
Influence of estrogen on sex differences in basal contraction. **a**, Comparison of fractional shortening in male, sham, OVX and OVX + 17β-estradiol (E2) myocytes, *n* = 12 cell/4 mice. **b**, Comparison of Ca^2+^ transient amplitude (Fura-2 ratio) in male, sham, OVX and OVX + E2 myocytes, n = 12 cell/4 mice. **c** The representative Ca^2+^ transient traces. **d**, Correlation between plasma 17β-estradiol and basal contraction amplitude, r = 0.8584, *P* < 0.05, *n* = 21. **e**, Effects of estrogen receptor agonists and antagonists on contraction. **f**, Effects of estrogen receptor agonists and antagonists on intracellular calcium amplitude. All data are presented as mean ± S.E.M. ^*^P < 0.05 and ^**^P < 0.01

Experiments were performed to investigate sex differences in left ventricular apical myocytes contraction and to determine the effect of E2 on contractility in FVB mice. At basal state, peak contractile amplitudes were significantly larger in Sham than in male and OVX myocytes (Fig. 1a). 1 h pretreatment with 1 nM E2 reversed the effects of OVX. 1 nM E2 was chosen as it is close to physiological concentration [25]. To further examine if similar differences existed in Ca^2+^ transient, myocytes were treated with Ca^2+^-sensitive dye 1 μM Fura-2 AM. Mean data showed that peak Ca^2+^ transient amplitude was larger in Sham compared to male and OVX myocytes (Fig. 1b). E2 treatment abolished the effects of OVX. Representative Ca^2+^ transient traces are shown in Fig. 1c. To test the correlation between plasma E2 and contractile amplitude, we matched plasma E2 levels with myocyte cell shortening. Spearman’s correlation analysis revealed that contraction amplitudes were positively and significantly correlated with plasma E2. (r = 0.8584; *P* < 0.05 (Fig. 1d). Collectively, these results show that contractility may be influenced by plasma E2 and that E2 may contribute to larger contraction amplitudes in female left ventricular apical myocytes compared to male. Since the effects of E2 observed here were acute, it is likely that they were mediated by non-genomic signaling. To determine the subtype of E2 receptor that mediated these effects, myocytes were pretreated with 1 nM E2, 100 nM G1, 100 nM G15, and 1 μM ICI 182,780 for 1 h. As shown in Fig. 1 (e and f), pretreatment of myocytes with ERα and ERβ antagonist did not abolish the effects of E2, but GPR30 antagonist (G15) did. Moreover, activation of GPR30 with G1 replicated the effects of E2 on contraction amplitude and Ca^2+^ transient amplitude suggesting that the acute effects of E2 were mediated by GPR30 via non-genomic signaling.

### Sex difference in *I*_*CaL*_ density of LV apical myocytes is modulated by E2

**Fig. 2:**
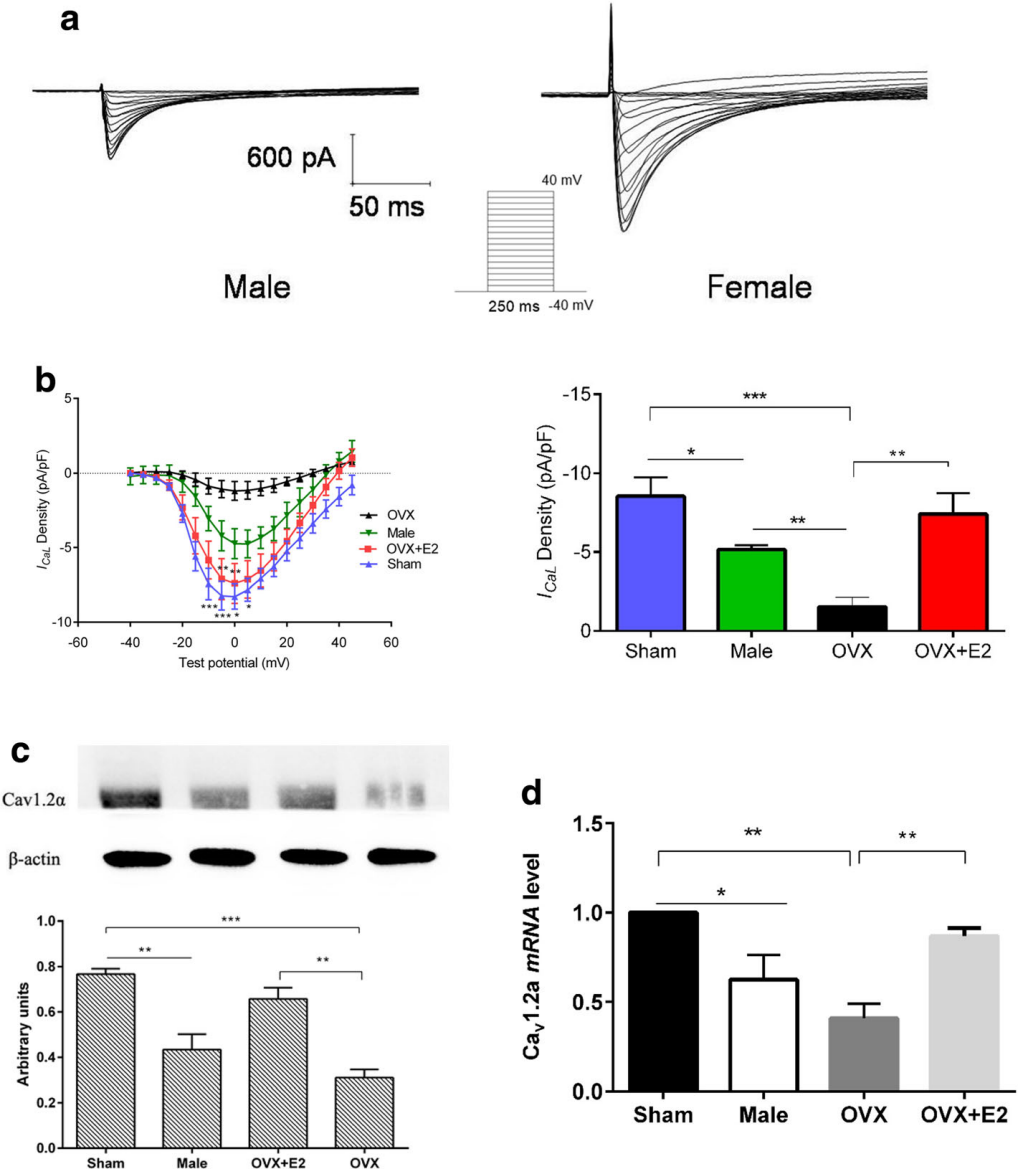
The effects of estrogen on sex differences in basal *I*_*CaL*_ density (**a**), Representative voltage-clamp protocol (shown in inset) and traces of *I*_*CaL*_ in male and female myocytes elicited by the voltage steps. **b** (left) current-voltage comparisons derived from the protocol shown in the inset in (**a**) for sham, male, OVX, and OVX + E2 myocytes. Peak *I*_*CaL*_ density is shown on the right, (*n* = 6–10 cells/4 mice). All presented data are mean ± S.E.M. (**c** and **d**). Estrogen modulates sex differences in basal *mRNA* and protein level of the L-type Ca^2+^ channel. **c** Comparisons of L-type Ca^2+^ channel protein levels in sham, male, OVX, and OVX + E2. (*n* = 3 mice). **d** L-type Ca^2+^ channel *mRNA* levels in sham, male, OVX, and OVX + E2. ^*^P < 0.05, ^**^P < 0.01, and ^***^P < 0.001. All data are presented as mean ± S.E.M

Cardiomyocytes were voltage-clamped using the protocol shown by the inset in Fig. 2a. Cell capacitance (pF) as a measurement of the membrane surface area was 163.4 ± 6.7, 147.2 ± 13.1, and 180.0 ± 12.8 pF for male, sham, and OVX, respectively (Table 1). Peak *I*_*CaL*_ density was higher in Sham myocytes compared to male and OVX, but E2 increased *I*_*CaL*_ density and restored it to normal levels. (Fig. 2b). These findings show that E2 increases *I*_*CaL*_ density and may be responsible for higher *I*_*CaL*_ density in female myocytes. To further examine whether the expression of LTCC at *mRNA* and protein differs between sexes and whether its expression is influenced by E2, we performed western blotting and real-time qPCR on isolated ventricular myocytes. Although the acute effects of E2 on functional parameters were tested after incubation for one hour, considering that the most mRNAs have a half-life of several hours, the effect of E2 on gene expression was tested after incubation for 5 h. LTCC *mRNA* and protein levels were markedly higher in sham compared to male and OVX myocytes. (Fig. 2 c and d). Incubation of OVX myocytes with 1 nM E2 for 5 h increased the expression of LTCC and reversed the effects of ovariectomy. Taken together, these data suggest that the sex differences in *I*_*CaL*_ density are due to differences in LTCC expression and may be modulated by E2.

### Basal cAMP levels are higher in sham compared to male and OVX myocytes

**Fig. 3:**
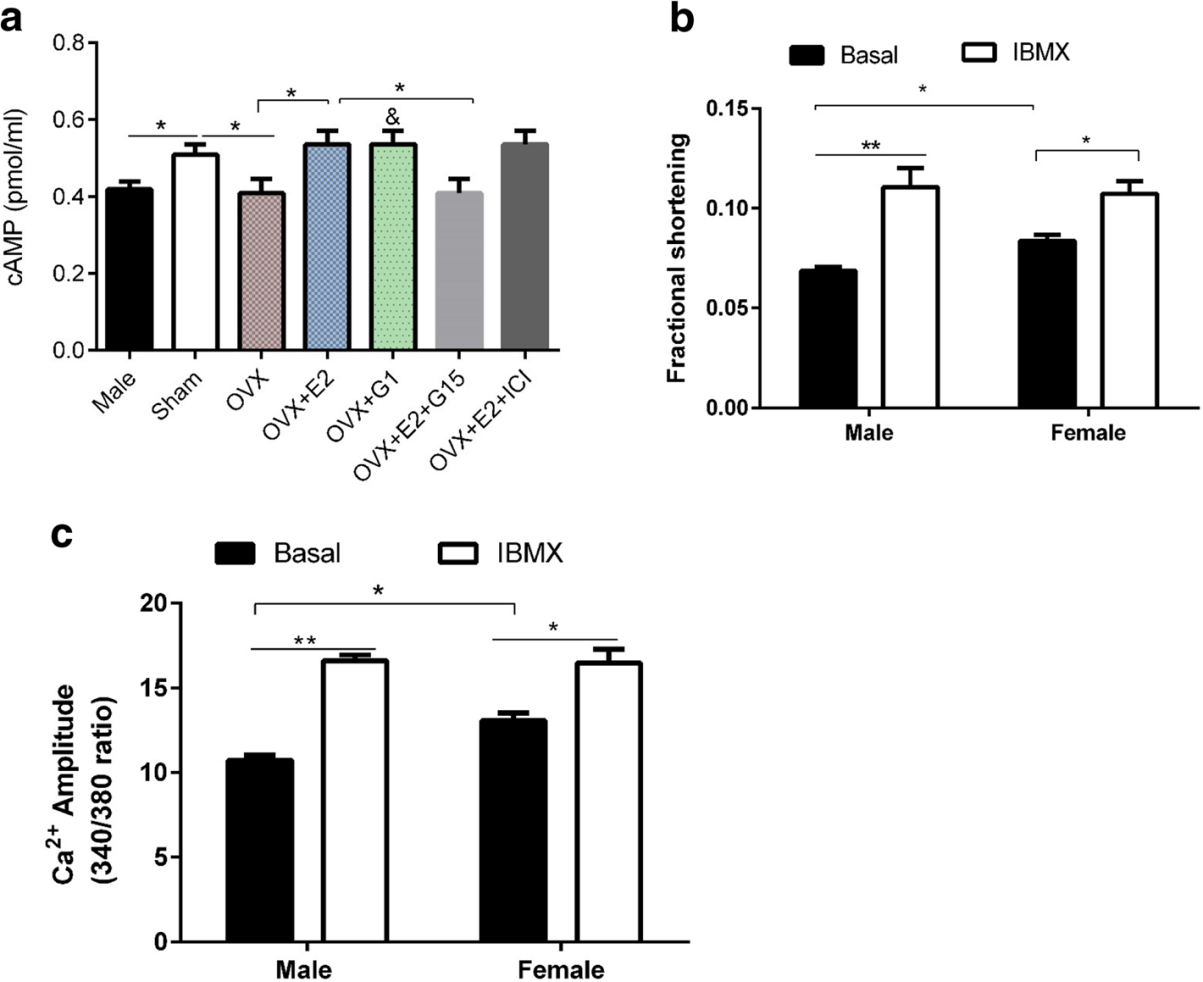
Inhibition of cAMP breakdown by 3-isobutyl-1-methylxanthine (IBMX) increased contraction amplitude and Ca^2+^ transient amplitude in both sexes and abolished the differences between them. **a** Basal cAMP levels among the groups and the effects of estrogen receptor agonists and antagonists on cAMP level (*n* = 5). **b**, Effect of IBMX on contraction amplitude in male and female (*n* = 5). **c** Effect of IBMX on Ca^2+^ transient amplitude. (*n* = 12 cells/6 mice). ^*^P < 0.05, and ^**^P < 0.01, ^&^*P* < 0.05 vs OVX. All data are presented as mean ± S.E.M

**Fig. 4:**
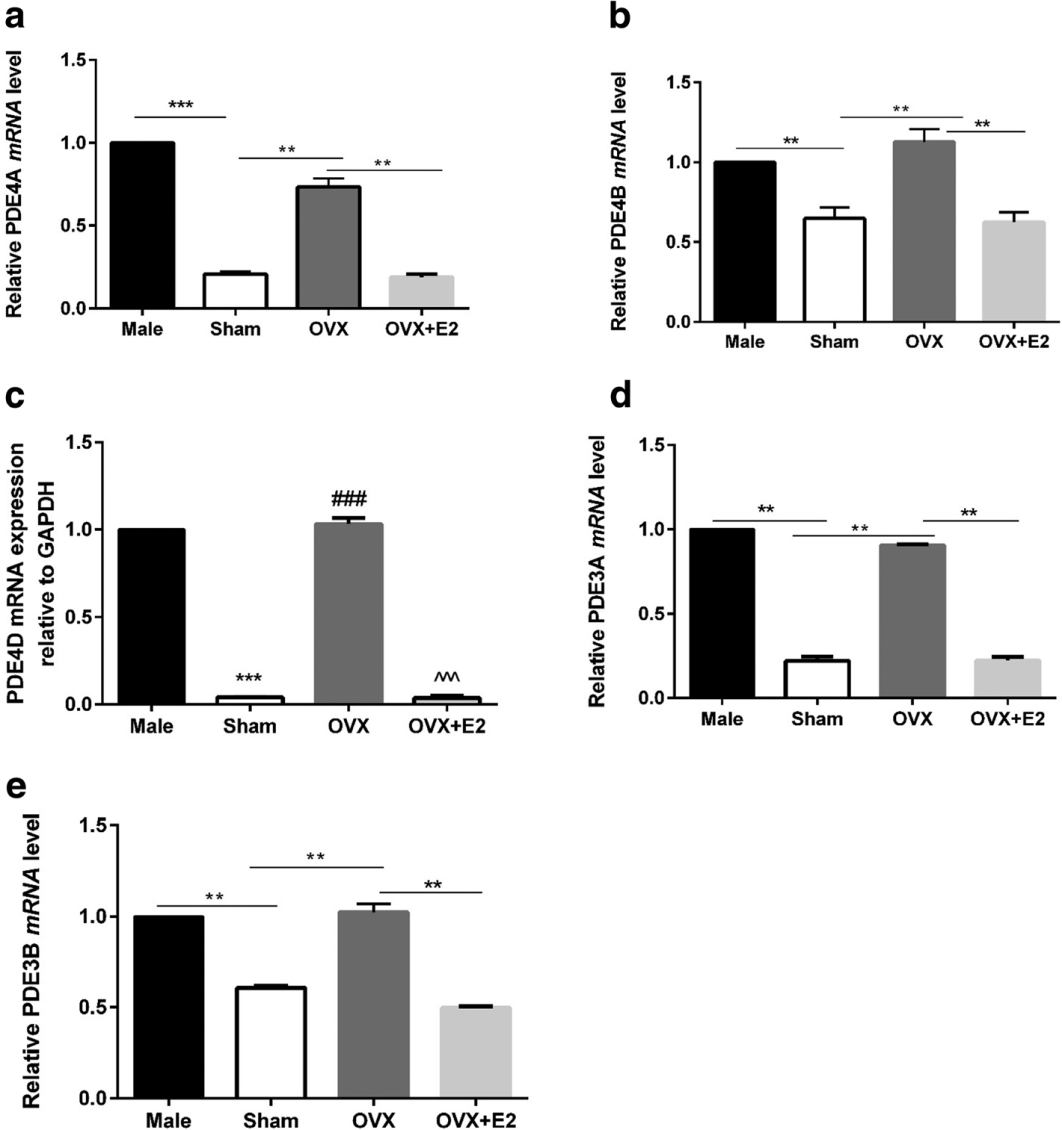
Sex differences in basal *mRNA* level of PDE3A, PDE3B, PDE4A, PDE4B, and PDE4D are mediated by E2. GAPDH levels were similar in all groups (**a**) PDE4A *mRNA* level in sham, male, OVX, and OVX + E2. (n = 3 mice). **b** PDE4B *mRNA* level in sham, male, OVX, and OVX + E2. (*n* = 3 mice). **c** PDE4D *mRNA* level in sham, male, OVX, and OVX + E2. (n = 3 mice). **d** PDE3A *mRNA* level in sham, male, OVX, and OVX + E2. (n = 3 mice). **e** PDE3B *mRNA* level in sham, male, OVX, and OVX + E2. (n = 3 mice). ^*^P < 0.05, ^**^P < 0.01, and ^***^P < 0.001. All data are presented as mean ± S.E.M

The cAMP-PKA-LTCC pathway regulates cardiomyocyte contraction. To elucidate whether sex differences in contraction and Ca^2+^ transient amplitude and *I*_*CaL*_ density could be explained by intracellular cAMP levels between male and female myocytes, we measured the basal level of cAMP using ELISA kit. The results are shown in Fig. 3a. Sham myocytes had higher cAMP levels compared to male and OVX. But OVX myocytes incubated with 1 nM E2 for 1 h had higher cAMP concentration compared to OVX. Pretreatment of myocytes with 1 nM E2, 100 nM G1, 100 nM G15, and 1 μM ICI 182,780 for 1 h revealed that ERα and ERβ antagonist (ICI 182,780) did not abolish the effects of E2 on cAMP level, but GPR30 antagonist (G15) did. Moreover, activation of GPR30 with G1 produced similar effects as E2 on cAMP level implying that the effects of E2 were mediated by GPR30 via non-genomic signaling. These results suggest that E2 increases cAMP in female myocytes. We then postulated that differential cAMP breakdown could underlie the sex differences in cAMP levels. To test this possibility, male and female myocytes were treated with non-selective PDE inhibitor, 3-isobutyl-1-methylxanthine (IBMX) 100 μM in DMSO for 10 min since PDE is responsible for cAMP degradation. The effects of IBMX were examined functionally by measuring contraction and Ca^2+^ transient amplitude. As shown in Fig. 3b-d, IBMX increased contraction and Ca^2+^ transient amplitude in both groups and eliminated the differences between them. These findings imply that cAMP breakdown is different between male and female mice myocytes. We further determined the *mRNA* levels of phosphodiesterases PDE 3A, 3B, 4A, 4B, and 4D in male and female myocytes and the effect of E2 on PDE expression. We found that the *mRNA* expression of all PDE subgroups was higher in male and OVX compared with Sham and OVX + E2 groups, respectively (Fig. 4). Collectively these observations indicated that (1) lower cAMP levels in male mice may be due to high cAMP breakdown by PDE compared to female and (2) OVX increased PDE *mRNA* levels while E2 decreased it.

### *mRNA* level of PLB, SLN, SERCA2a, and RyR2 in LV apical myocytes are modulated by E2

**Fig. 5:**
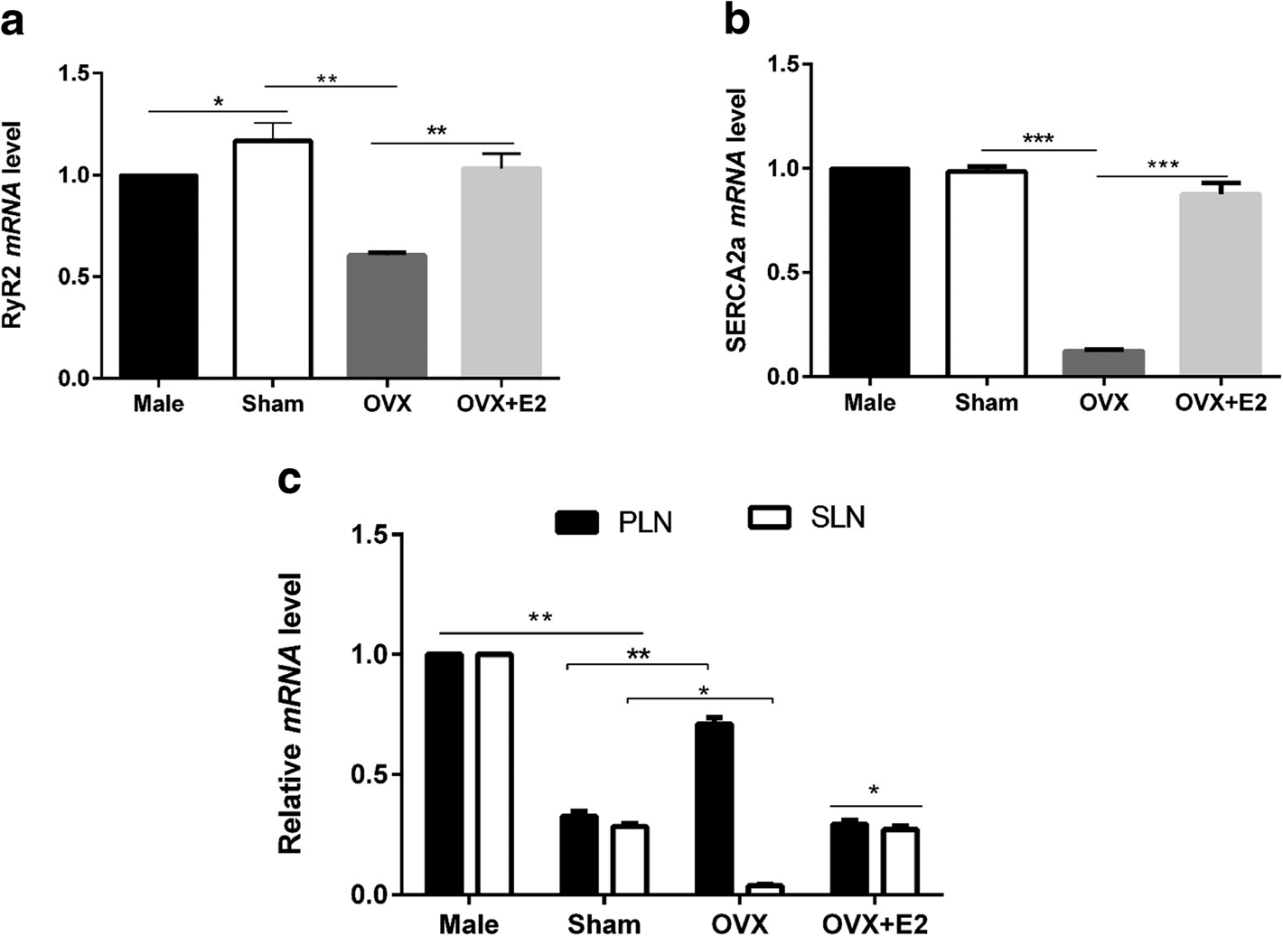
Sex differences in basal *mRNA* expression of PLB, SLN, SERCA2a, and RyR2 are modulated by E2. GAPDH levels were similar in all groups (**a**) Relative RyR2 *mRNA* levels normalized to GAPDH *mRNA* in sham, male, OVX, and OVX + E2. (n = 3 mice). **b** Relative SERCA2a *mRNA* levels normalized to GAPDH *mRNA* in sham, male, OVX, and OVX + E2. (n = 3 mice). **c** Relative PLB and SLN *mRNA* levels normalized to GAPDH *mRNA* in sham, male, OVX, and OVX + E2. (n = 3 mice). ^*^P < 0.05, ^**^P < 0.01, and ^***^P < 0.001. All data are presented as mean ± S.E.M

We also compared the expression of other proteins involved in regulation of intracellular Ca^2+^ transients between sexes, and whether they are regulated by E2. *mRNA* levels of PLB, SLN, SERCA2a, and RyR2 were determined by real-time qPCR. Figure 5a shows that RyR2 expression was higher in sham myocytes and OVX + E2 compared with male and OVX myocytes, respectively. These data suggest that E2 may be responsible for the sex difference in RyR2 levels. High levels of RyR2 are associated with high Ca^2+^ release from sarcoplasmic reticulum (SR). There was no significant difference in SERCA2a *mRNA* levels between male and sham (Fig. 5b). However, OVX decreased SERCA2a *mRNA* level while E2 reversed this effect. SLN and PLB regulate cardiomyocyte contraction through their inhibitory effects on SERCA2a and they are targeted by cAMP-PKA. As shown in Fig. 5c, both SLN and PLB were lower in Sham compared to male. Interestingly, OVX increased PLB while it decreased SLN compared to Sham. The effects of OVX were reversed by E2 treatment for 5 h.

### E2 modulates sex responses to stress in LV apical myocytes

**Fig. 6:**
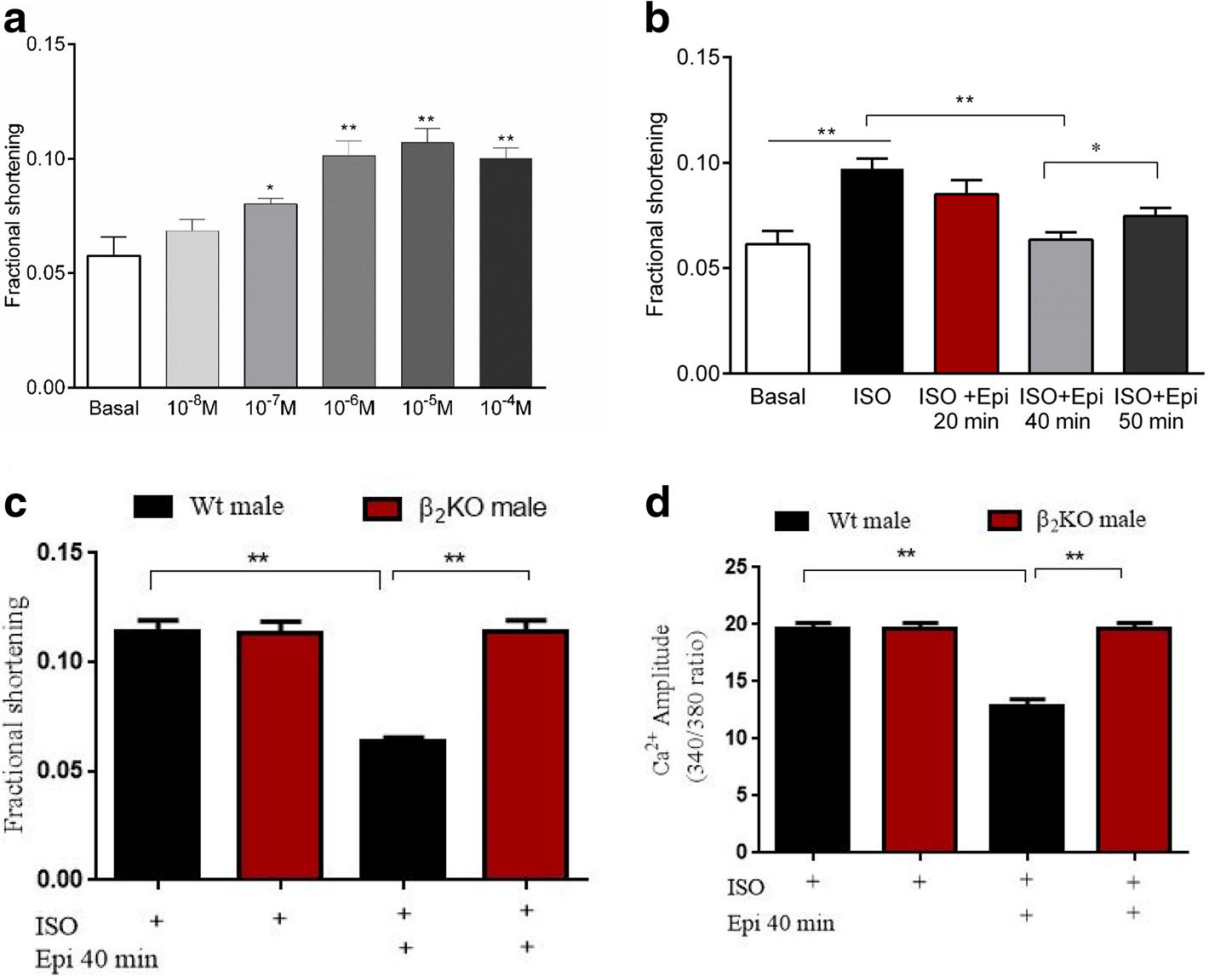
β_2_AR-induced stress model in male myocytes. **a**, Contractile responses to β-adrenergic receptor stimulation by increasing concentration of isoproterenol (ISO) (*n* = 10 cells/4 mice) (**b**), Effect of epinephrine (Epi) stress on cardiac contraction. (*n* = 10 cells/4 mice). **c** Effects of stress on contraction amplitude abolished by β_2_AR knockout (β_2_KO) n = 10 cells/4 mice. **d** Effects of stress on Ca^2+^ transient amplitude were abolished by β_2_KO, n = 10 cells/4 mice. ^*^P < 0.05, ^**^P < 0.01, and ^***^P < 0.001. All data are presented as mean ± S.E.M

**Fig. 7:**
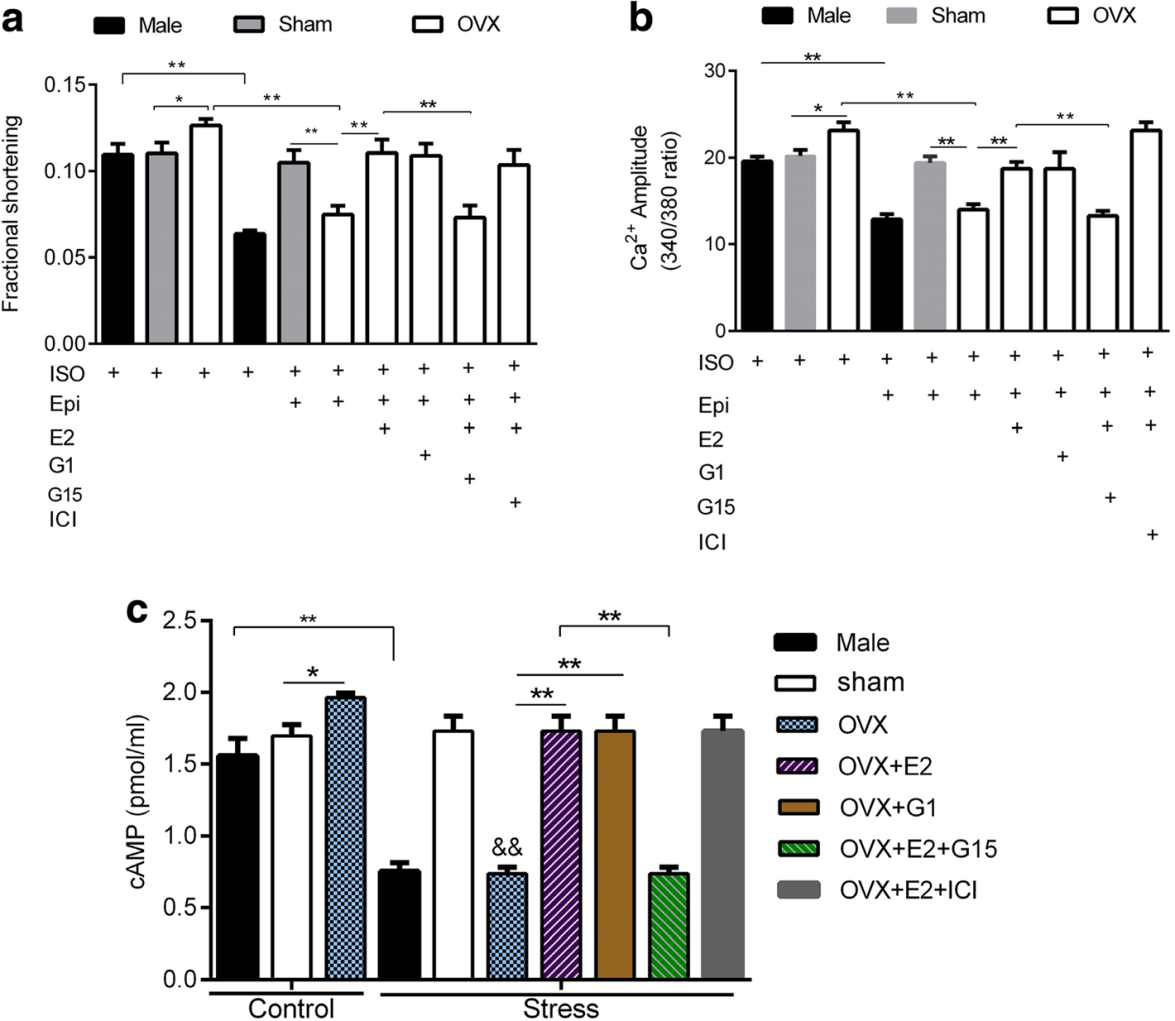
Contractile response to βAR stimulation and stress in cardiomyocytes and the influence of estrogen receptor agonists and antagonists. **a** Contractile responses to stress in male, sham, OVX, and OVX + E, in the presence or absence of estrogen receptor agonists and antagonists. **b** Corresponding Ca^2+^ transient amplitude in male, sham, OVX, and OVX + E in the presence or absence of estrogen receptor agonists and antagonists (n = 10 cells/7 mice). **c** Effect of ISO-stimulation (control) and Epi (stress) on cAMP in the presence or absence of estrogen receptor agonists and antagonists (n = 10 cells/7 mice). ^*^P < 0.05, ^**^P < 0.01, ^&&^*P* < 0.01 vs control OVX. All data are presented as mean ± S.E.M

On the basis of β_2_AR signal trafficking, we developed an acute in vitro stress model in male LV cardiomyocytes according to a protocol established previously [23]. A pilot experiment was performed to determine the β-adrenergic receptor (βAR)-response to isoproterenol (ISO), a non-specific β_1_AR/β_2_AR agonist. As shown in Fig. 6a, incremental increases in ISO concentration elicited corresponding increases in myocyte shortening amplitude. Optimal amplitude shortening was observed at 1 μM ISO and this concentration was chosen for subsequent βAR stimulation. Stress was induced by incubating myocytes with epinephrine (Epi), which has a higher affinity for β_2_AR. Contractile responses to stress were evaluated at 20, 40, and 50 min using ISO. As shown in Fig. 6b, contractility was significantly decreased at 40 min of Epi incubation compared to the control group. The decline in contractility tended to recover at 50 min showing a recovery phase. The β_2_AR-mediated stress was confirmed using β_2_AR-gene knockout (β_2_KO) cardiomyocytes. The effects of stress on contraction and Ca^2+^ transient amplitude were eliminated by β_2_AR gene knockout (Fig. 6c and d). To test if E2 modulates sex differences in stress responses, apical LV myocytes from male, Sham, OVX, and OVX + E2 (myocytes pretreated with E2 after isolation for 1 h) were incubated with Epi for 40 min and their response to ISO challenge was examined. As shown in Fig. 7 a and b, there were no significant sex differences in contraction and Ca^2+^ amplitude elicited by ISO, but OVX myocytes had higher amplitudes compared to Sham implying that OVX increased sensitivity to ISO.

During stress, contraction amplitude and peak Ca^2+^ transient were decreased only in wild-type (Wt) male and OVX groups but not in Wt Sham myocytes (Fig. 7a and b). Pre-treatment of OVX myocytes with 1 nM E2 and G1 (100 nM) increased contraction and Ca^2+^ transient amplitude and restored the response to ISO. Moreover, pretreatment of myocytes with GPR30 antagonist (100 nM, G15) eliminated the effects of E2, but pretreatment with ERα and ERβ antagonist (1 μM, ICI 182,780) did not abolish the effects of E2. These datasets imply that GPR30 mediated the acute effects of E2 via non-genomic signaling. We further tested whether intracellular cAMP concentration was altered during stress. Similarly, we found that stress decreased cAMP concentration only in Wt male and OVX groups but not in Wt Sham myocytes. Moreover, 1 nM E2 and 100 nM G1 reversed the effects of OVX (Fig. 7c). Treatment of myocytes with G15 abolished the effect of E2 but co-treatment with CI 182,780 did not. These findings suggest that the β_2_AR-induced stress decreased cAMP thereby reducing contraction and Ca^2+^ transient amplitude. In addition, Sham myocytes were resistant to stress which may be attributed to E2 protection.

### E2 compensates for the loss of function resulting from a β_2_AR-gene knockout in female myocyte

**Fig. 8:**
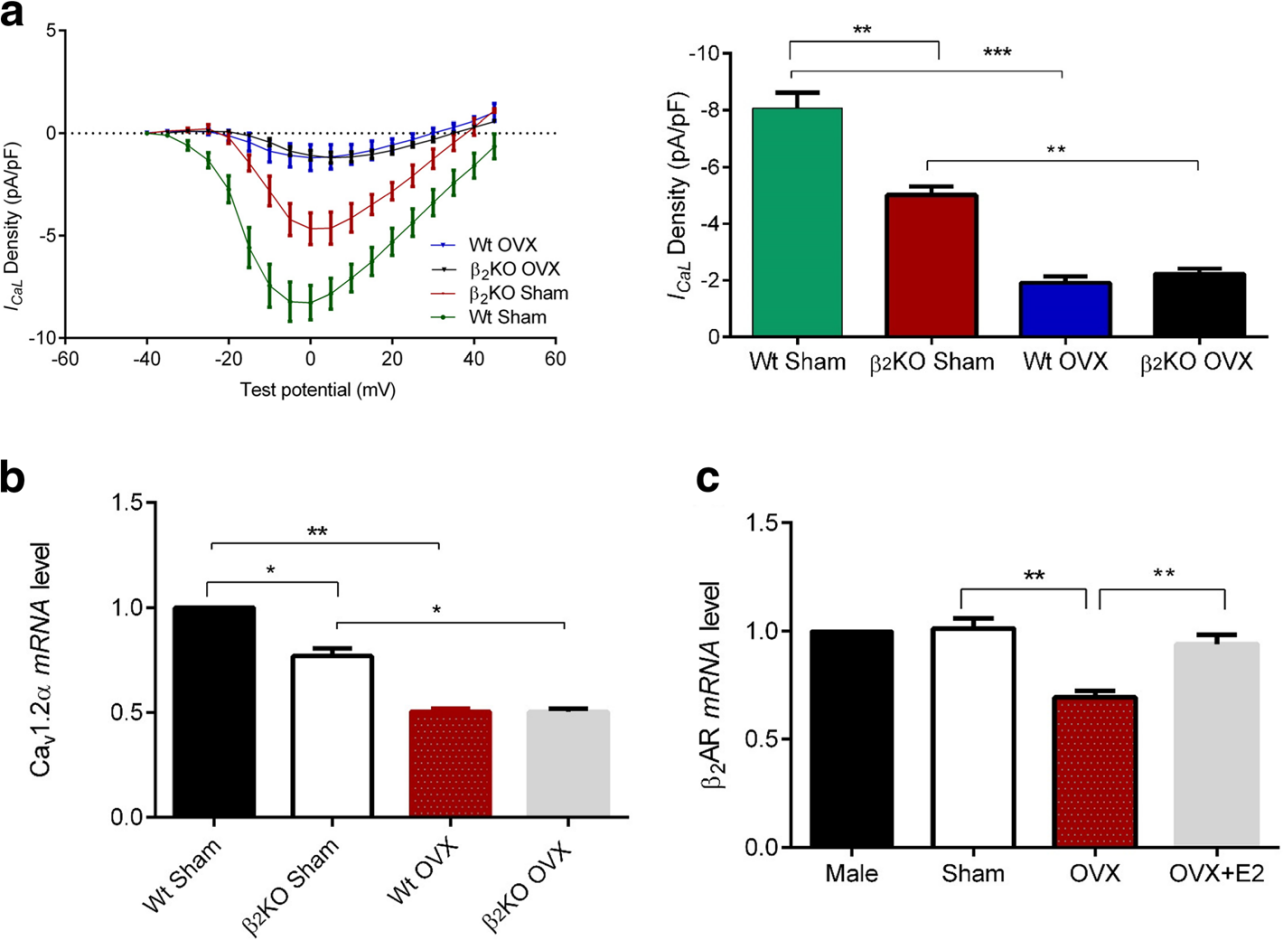
Estrogen compensates for the loss of function resulting from β_2_AR-gene knockout in female LV apical myocytes. **a** current-voltage comparisons among Wt sham, Wt male, OVX, and OVX + E2 myocytes. (n = 6–10 cells/4 mice). **b** Effect of β_2_AR on *mRNA* expression of the L-type Ca^2+^ channel depends on the E2 environment. (n = 3 mice). **c** Influence of E2 on β_2_AR *mRNA* level. (n = 3 mice). ^*^P < 0.05, ^**^P < 0.01, ^***^P < 0.001. All data are presented as mean ± S.E.M

As further illustrated in Fig. 8a, *I*_*CaL*_ density was smaller in β_2_AR KO Sham compared to Wt Sham myocytes, but it was not significantly different between Wt OVX and β_2_AR KO OVX myocytes. Similarly, LTCC *mRNA* level was decreased in β_2_AR KO Sham myocytes compared to Wt Sham (Fig. 8b). Ovariectomy decreased LTCC *mRNA* level and eliminated the differences between wild-type Sham and β_2_KO Sham myocytes. These results indicate that the decrease of LTCC expression and *I*_*CaL*_ density in female β_2_KO myocytes was depended on the E2 environment. Furthermore, we found that although there were no sex differences in *mRNA* level of β_2_AR, OVX decreased it while E2 abolished this effect (Fig. 8c). These results show that the effects of β_2_AR on LTCC may be influenced by E2.

## Discussion

In this study, we investigated sex differences in basal contractility and responses to catecholamine-induced stress in LV apical myocytes. We found that (1) *I*_*CaL*_ density, contraction and Ca^2+^ transient amplitudes were larger in Sham compared to male and OVX myocytes at basal state. E2 reversed the effects of OVX on these parameters. (2) Basal cAMP concentration was higher in Sham myocytes compared to male and OVX; this was due to a lower cAMP breakdown in Sham myocytes which had lower *mRNA* levels of PDE 3A, 3B, 4A, 4B, and 4D compared with male and OVX. The effects of OVX on PDE expression were abolished by E2 treatment. Moreover, inhibition of cAMP breakdown by 100 μM IBMX increased contraction and Ca^2+^ transient amplitude in both sexes and canceled differences between them. (3) In stress state, cAMP concentration, contraction amplitude, and peak Ca^2+^ transient were decreased in male and OVX groups but not in Sham myocytes. Treatment of OVX myocytes with E2 in increased cAMP levels, contraction, and Ca^2+^ transient amplitude. (4) Pretreatment of myocytes with ERα and ERβ antagonist ICI 182,780 (1 μM) did not abolish the effects of E2, but GPR30 antagonist G15 (100 nM) did. Moreover, activation of GPR30 with G1 (100 nM) replicated the effects of E2 on cAMP, contraction and Ca^2+^ transient amplitudes suggesting that the acute effects of E2 were mediated by GPR30 via non-genomic signaling. Collectively, these results show that E2 plays a key role in sex contractile differences in LV apical myocytes.

Male and female hearts have well-known contractile differences. Here, differences in basal contraction (in absence of agonist) and response to stress were observed. A summary of previous studies comparing sex differences is presented in Table 3. Similar to some studies [26–28], female myocytes had a larger contraction and Ca^2+^ transient amplitudes compared to male myocytes (Fig. 1a-c). In contrast, Farrell et al. [5] found that female rat myocytes had a smaller contraction and Ca^2+^ transient amplitudes than males. This discrepancy may be explained by the experimental model (whole ventricular myocytes versus or LV apical myocytes) or species differences. In addition, in their study, rats were age-matched. But in this study, mice were weight-matched, and therefore, it is likely that male mice were younger than female considering that the growth rate of female mice is much slower than males [29]. Hence, as reported by Howlett et al., contraction in aged female is higher compared to younger male [6]. Other likely causes of the discrepancy are the experimental conditions such as (1) contraction and Ca^2+^ transient recorded at 37 °C versus 23 °C used in this study. Temperature would affect the activity of ion channels of E-C coupling [30]. (2) In their study, contractions and Ca^2+^ transients were activated simultaneously with 250-ms test steps to different potentials. Here, field stimulation with action potential were used. These two methods were found to produce different level of fractional shortening, being higher in stimulation by voltage-clamp steps [5]. Equally interesting is that Parks et al. [31] found that Ca^2+^ transient was smaller in female than in male C57BL/6 mice with no differences in *I*_*CaL*_ density between the sexes. The discrepancy observed here may arise from several factors. In their experiments, global ventricular myocytes were investigated while in our study, only apical myocytes of the left ventricle were studied. This presents a major source of discrepancy considering that apical versus basal variations in *I*_*CaL*_ density have been reported in rabbit hearts [32]. In addition, differential distribution of βAR receptors in the left ventricle (i.e. β_2_AR > β_1_AR at the apex and β_1_AR > β_2_AR at the base [17]) implies that myocytes from these regions may respond differently to stimulation. However, it remains to be determined whether there are sex differences in βAR distribution in the left ventricle regions.

**Table 3:** A summary of studies comparing parameters of cardiac contraction between sexes

Species	Heart preparation	Contraction of female compared to male	Ca^2+^transient	*I*_*CaL*_density	Stimulation frequency (Hz)	Recording temperature	External Ca^2+^concentration	Ref.
Rat	Whole ventricular myocytes	Lower	Lower	Similar	1	37 °C	1.8 mM	[5]
Rat	Whole ventricular myocytes	Lower	Similar	Similar	2–4 Hz	37 °C	1.8 mM	[6]
Rat	left ventricle myocytes	Lower	lower	–	1 Hz	25 °C	0.5–2 mM	[40]
Rat	skinned ventricular papillary muscle fiber	Higher	–	–	0.5 Hz	Room temp.	1.5 mM	[26]
Rat	Whole ventricular tissue	Higher	–	Higher	0.8 Hz	–	1 mM	[27]
Rat	skinned atrial fibers	Higher	–	–	0.4 Hz	32 °C	1–7 mM	[28]
B6SJLF1/J mice	Whole ventricular tissue	Similar	Similar	–	2 Hz	37 °C	1 mM	[38]
Female dogs	Epicardial Cells, endocardium, mid-myocardium	–	–	Higher	2 Hz	37 °C	2 mM	[35]
Guinea-pigs	Left ventricular myocytes	–	–	Higher	0.5 Hz	37 °C	1 mM	[36]
Rabbit	Base left ventricular myocytes	–	–	Higher	5 Hz		2.5 mM	[32]

Short-term OVX (21 days) decreased the contractions and Ca^2+^ transients and pretreatment of myocytes with E2 abolished these effects implying that they were mediated by E2. Moreover, pretreatment of OVX myocytes with GPR30 antagonist G15 (100 nM) abolished the effects of E2, whereas ERα and ERβ antagonist ICI 182,780 (1 μM) did not. Treatment of OVX myocytes with G1 (100 nM) replicated the effects of E2 on contraction and Ca^2+^ transient amplitudes suggesting that the acute effects of E2 were mediated by GPR30 via non-genomic signaling. In contrast, in guinea pigs, long-term OVX increased Ca^2+^ transient [33]. One possible mechanism to explain this discrepancy is that loss of E2 may have time-dependent effects on the transcription of genes involved in E-C coupling. Nevertheless, they found that OVX decreased fractional shortening in vivo which agrees to our in vitro results. It was previously shown that plasma E2 varies with the estrous cycle in murine [22]. Therefore, we explored the correlation between myocyte contraction and plasma E2 using Spearman’s correlation test. As illustrated in Fig. 1, fractional shortening was positively correlated with plasma E2, suggesting that E2 may positively affect contractility. Although we did not confirm whether plasma E2 correlates with intracellular E2 levels in cardiomyocytes, E2 has been shown to affect heart autonomic functions such as heart rate [8]. In addition, James et al. found that *I*_*CaL*_ varies with estrous cycle in guinea pigs [34]. Hence, it is likely that plasma E2 affects contractility.

LTCC plays an important role in the regulation of contraction and intracellular Ca^2+^. The size of *I*_*CaL*_ is proportional to the Ca^2+^ transient amplitude [13], but studies have been inconsistent regarding the size of *I*_*CaL*_ density between sexes. Hence, we further investigated whether sex differences in contraction and Ca^2+^ transient were due to different levels of *I*_*CaL*_ density between sexes. Consistent with previous studies, our data demonstrated that *I*_*CaL*_ density was larger in Sham myocytes compared to male [27, 35, 36], suggesting that the larger contraction in female compared to male may be caused by larger *I*_*CaL*_ density in female although other studies [5, 6, 37–39] did not find sex differences in *I*_*CaL*_ density. This discrepancy could be due to inter-strain/species differences or *I*_*CaL*_ recording conditions (see Table 3). OVX decreased *I*_*CaL*_ density which was reversed by E2 (Fig. 2b). This implies that E2 modulated sex differences in *I*_*CaL*_ density. Moreover, we performed western blot and Real-time qPCR on LV apical cardiomyocytes from between sexes. As illustrated in Fig. 2 c and d, LTCC protein and *mRNA* levels were higher in Sham myocytes than in male myocytes suggesting that high *I*_*CaL*_ in Sham compared to male was due to the high number of LTCC. Previously, it was found that long-term OVX (8 months) decreased LTCC protein and *I*_*CaL*_ density in mice [41]. However, in this study, short-term OVX (14–21 days) decreased LTCC *mRNA*, protein, and *I*_*CaL*_ density, which were reversed by 1 nM E2 treatment. In agreement with these findings, treatment of female cardiomyocytes derived from induced pluripotent stem cells (iPS-CM) with 1 nM E2 increased LTCC *mRNA* level and *I*_*CaL*_ [42]. Similarly, the expression of LTCC protein in rat heart ventricle was found to be higher in female than male [43]. Recently, using H9C2 and rat myocytes, Yang et al. demonstrated that treatment with E2 enhanced *I*_*CaL*_ and Cav1.2α1C expression through plasma membrane-bound ER. This finding is in agreement with our study [44].

The cAMP-PKA-LTCC pathway is an important component of cardiomyocyte contraction. To determine whether there are sex differences in intracellular cAMP levels, LV apical myocytes were subjected to cAMP ELISA test. As shown in Fig. 3a, Sham myocytes had higher levels of cAMP compared to male. OVX decreased cAMP while E2 reversed these effects. Our results contrast with those reported by Parks et al. [45]. In their study, contraction and Ca^2+^ transient amplitude were higher in OVX than Sham and but no differences were found in basal cAMP between Sham and OVX ventricular myocytes. The differences noted here may be partly due to the type of myocytes used i.e. global ventricular myocytes versus left ventricular apical myocytes in our study. In addition, they investigated the long-term effects of OVX (8 months) which may be different from the short-term OVX (21 days) effects in this study. Taken together, the cAMP results supported the findings that contraction and Ca^2+^ transient were higher in Sham than in male myocytes.

We then postulated that differential cAMP breakdown may be responsible for the sex differences in cAMP levels. The effects of non-selective PDE inhibitor (IBMX) were determined functionally in terms of contraction and Ca^2+^ transient amplitude. As shown in Fig. 3b-d, IBMX increased contraction and Ca^2+^ transient amplitude in male and female myocytes and eliminated the sex differences. These findings imply that cAMP breakdown is different between sexes at the basal state. Further analysis of *mRNA* level of PDEs (PDE 3A, 3B, 4A, 4B, and 4D [46]) revealed that *mRNA* levels of these PDEs were higher in male and OVX LV apical myocytes than Sham and OVX + E2 groups (Fig. 4). Collectively, these observations imply that (1) lower cAMP levels in male were due to high cAMP breakdown by PDE compared to female and (2) E2 modulates the *mRNA* level of PDE. These observations differ from those reported by Parks et al. who found that long-term OVX increased expression of only PDE4A [45]. They also found that cAMP was lower in female due to higher PDE4B than male [31] and no sex differences were found for PDE3 and 4 families. These discrepancies are possibly due to model differences described above.

We further compared the expression of PLB, SLN, SERCA2a, RyR2 in male, Sham and OVX myocytes at the gene level. Figure 5 shows that RyR2 *mRNA* expression was higher in sham myocytes than male while both SLN and PLB were higher in male compared to Sham. OVX decreased RyR2 and SLN *mRNA* level but it increased PLB expression and the effects of OVX were reversed by E2. These data indicate that sex differences exist in the expression of PLB, SLN and RyR2, and their expression in female myocytes is regulated by E2. High levels of RyR2 are associated with high Ca^2+^ release from sarcoplasmic reticulum [47]. Consequently, a lower level of RyR2 in male myocytes may cause smaller SR Ca^2+^ release and hence smaller Ca^2+^ transient and contraction amplitude compared to female. Both SLN and PLB are phosphorylated by PKA [48]. The higher SLN and PLB in male myocytes compared to Sham (Fig. 5c) would result in strong superinhibition of SERCA2 and eventually suppress cardiac contractility [49]. Interestingly, no sex differences were observed in the expression of SERCA2a Fig. 5b). But OVX decreased SERCA2a *mRNA* level compared to Sham. Furthermore, OVX increased PLB while it decreased SLN compared to Sham. These effects of OVX were reversed by 1 nM E2 treatment. Even though the phosphorylation state of these proteins was not examined, their activity is partly dependent on their expression level. Taken together, these results further suggest that low SLN and PLB, high RyR2 in female than male may contribute to a higher contraction in female myocytes compared to male.

Stress-induced cardiomyopathy is predominant among aged postmenopausal women compared to men and premenopausal women [50]. In a previous study [23], a 20-min exposure of male rat cardiomyocytes to Epi decreased contractile response to ISO due to signal trafficking from β_2_AR-Gs to β_2_AR-Gi. Here, we established the stress model in left ventricular apical mice myocytes. The results showed that the β_2_AR-mediated decline in contraction was observed at 40 min (Fig. 6b) and was absent in β_2_KO myocytes. The difference in the time frame of Epi effects may be explained by differential βAR coupling to Gs and Gi as reported in different species [51]. This study also revealed sex-specific responses to stress. Epi treatment decreased contraction and Ca^2+^ transient amplitude only in Wt male and OVX myocytes but not in Wt sham and OVX + E2 myocytes (Fig. 7a). Furthermore, stress has similar effects on cAMP levels (Fig. 7c). Our data implied that E2 acted through GPR30 to suppress the effects of stress which is in line with our previous study [52]. These findings show that female myocytes with normal E2 levels are protected against β_2_AR-mediated stress. This is consistent with a previous study [19], in which E2 modulated cardiac responses to emotional stress. Figure 8 a and b shows that lack of ovarian hormones due to OVX influenced the effects of β_2_AR on the LTTC *mRNA* and *I*_*CaL*_ density. β_2_KO Sham myocytes had decreased *I*_*CaL*_ density and LTCC *mRNA* level compared to wild-type female. Interestingly, OVX decreased both LTCC *mRNA* level and *I*_*CaL*_ density and eliminated the differences between wild-type Sham and β_2_KO Sham myocytes. These results show that estrogen compensates for the loss of function resulting from β_2_AR-gene knockout in female LV apical myocytes. Furthermore, OVX decreased β_2_AR *mRNA* while E2 normalized its expression (Fig. 8c). However, it should be noted that OVX leads to the loss of all ovarian hormones, besides estrogen. These findings are in line with our previous report that β_2_AR-mediated cardioprotection was depended on the E2 status [53].

## Conclusion

This study shows, for the first time, that sex contractile differences exist in LV apical myocytes which are mediated by E2 through modulation of gene transcription in the cAMP-PKA-LTCC pathway. We also reveal that E2 confers cardioprotection against β_2_AR-derived signals during cardiac stress. The differential cAMP breakdown in left ventricular apical myocytes (between sexes) contributes to differences in contraction and responses to stress. Equally important are the sex differences in the expression of RyR2, PLB and SLN. The data presented here have revealed new concepts that are likely to be important for additional investigations into the roles of β_2_AR and E2 (and its receptors) in cardiac contraction. Given the current findings at single myocytes level, we are currently carrying out further studies at tissue and organ levels to validate these results and compare contraction in basal left ventricular myocytes. It will be interesting to determine the transcriptional mechanism by which E2 influenced the expression of the genes studied in this work. Nonetheless, this study provides important findings on the role of estrogen in cardiac physiology and sex-dependent cardiomyopathies.

## Abbreviations

AC: Adenylate cyclase
cAMP: Cyclic adenosine monophosphate
E2: 17β-estradiol
Epi: Epinephrine
ER: Estrogen receptor
ERα: Estrogen receptor α
ERβ: Estrogen receptor β
FVB: Mice Friend leukemia virus B
GAPDH: Glyceraldehyde 3-phosphate dehydrogenase
Gi: Inhibitory G protein
GPR30: G protein-coupled receptor 30
Gs: Stimulatory G protein
IBMX: 3-isobutyl-1-methylxanthine
I_Ca-L_: L-type calcium current
ISO: Isoprenaline
KO: Gene Knockout mice
LTCC: L-type calcium channel
LV: Left ventricle
mRNA: Messenger RNA
OVX: Ovariectomized female
PBS: Phosphate-buffered saline
PDE: Phosphodiesterase
PI3K: Phosphoinisitide 3-kinase
PKA: Protein kinase A
PLB: Phospholamban
RyR2: Ryanodine receptor 2
SCM: Stress cardiomyopathy
SERCA2a: Sarcoplasmic reticulum Ca^2+^ ATPase 2a
Sham: Sham operated mice
SLN: Sarcolipin
SR: Sarcoplasmic reticulum
Wt: Wild-type
β_1_AR: β_1_-adrenergic receptor
β_2_AR: β_2_-adrenergic receptor
β-ARs: βeta-adrenergic receptors

## References

[1] Bening C, Weiler H, Vahl C-F (2013). Effects of gender, ejection fraction and weight on cardiac force development in patients undergoing cardiac surgery--an experimental examination. J Cardiothorac Surg.

[2] James AF, Choisy SCM, Hancox JC (2007). Recent advances in understanding sex differences in cardiac repolarization. Prog Biophys Mol Biol.

[3] Verkerk AO, Wilders R, de Geringel W, Tan HL (2006). Cellular basis of sex disparities in human cardiac electrophysiology. Acta Physiol (Oxf)..

[4] Zhu B, Liu K, Yang C, Qiao Y, Li Z (2016). Gender-related differences in β-adrenergic receptor-mediated cardiac remodeling. Can J Physiol Pharmacol.

[5] Farrell SR, Ross JL, Howlett SE (2010). Sex differences in mechanisms of cardiac excitation-contraction coupling in rat ventricular myocytes. Am J Physiol Heart Circ Physiol.

[6] Howlett SE (2010). Age-associated changes in excitation-contraction coupling are more prominent in ventricular myocytes from male rats than in myocytes from female rats. Am J Physiol Heart Circ Physiol.

[7] Machuki J.O., Zhang H.Y., Harding S.E., Sun H. (2017). Molecular pathways of oestrogen receptors and β-adrenergic receptors in cardiac cells: Recognition of their similarities, interactions and therapeutic value. Acta Physiologica.

[8] McKinley PS, King AR, Shapiro PA (2009). The impact of menstrual cycle phase on cardiac autonomic regulation. Psychophysiology.

[9] Zoma WD (2004). baker RS, Clark KE. Effects of combined use of sildenafil citrate (Viagra) and 17beta-estradiol on ovine coronary and uterine hemodynamics. Am J Obstet Gynecol.

[10] Cao X, Zhou C, Chong J (2015). Estrogen resisted stress-induced cardiomyopathy through increasing the activity of β_2_AR-Gαs signal pathway in female rats. Int J Cardiol.

[11] Wu Q, Zhao Z, Sun H, Hao Y, Yan C, Gu S (2008). Oestrogen changed cardiomyocyte contraction and beta-adrenoceptor expression in rat hearts subjected to ischaemia-reperfusion. Exp Physiol.

[12] Chen-Izu Y, Xiao RP, Izu LT (2000). G(i)-dependent localization of beta (2)-adrenergic receptor signaling to L-type ca (2+) channels. Biophys J.

[13] Bers DM (2008). Calcium cycling and signaling in cardiac myocytes. Annu Rev Physiol.

[14] Xiang Y, Naro F, Zoudilova M, Jin S-LC, Conti M, Kobilka B (2005). Phosphodiesterase 4D is required for beta2 adrenoceptor subtype-specific signaling in cardiac myocytes. Proc Natl Acad Sci U S A.

[15] Mika D, Bobin P, Pomerance M (2013). Differential regulation of cardiac excitation-contraction coupling by cAMP phosphodiesterase subtypes. Cardiovasc Res.

[16] Leroy J, Abi-Gerges A, Nikolaev VO (2008). Spatiotemporal dynamics of beta-adrenergic cAMP signals and L-type Ca2+ channel regulation in adult rat ventricular myocytes role of phosphodiesterases. Circ Res.

[17] Lyon AR, Rees PSC, Prasad S, Poole-Wilson PA, Harding SE (2008). Stress (Takotsubo) cardiomyopathy--a novel pathophysiological hypothesis to explain catecholamine-induced acute myocardial stunning. Nat Clin Pract Cardiovasc Med.

[18] Nef HM, Mollmann H, Kostin S (2007). Tako-Tsubo cardiomyopathy intraindividual structural analysis in the acute phase and after functional recovery. Eur Heart J.

[19] Ueyama T, Ishikura F, Matsuda A (2007). Chronic estrogen supplementation following ovariectomy improves the emotional stress-induced cardiovascular responses by indirect action on the nervous system and by direct action on the heart. Circ J.

[20] Meyer S, Brouwers FP, Voors AA (2015). Sex differences in new-onset heart failure. Clin Res Cardiol.

[21] Zhou YY, Wang SQ, Zhu WZ (2000). Culture and adenoviral infection of adult mouse cardiac myocytes methods for cellular genetic physiology. Am J Physiol Heart Circ Physiol.

[22] McLean AC, Valenzuela N, Fai S, Bennett SAL (2012). Performing vaginal lavage, crystal violet staining, and vaginal cytological evaluation for mouse estrous cycle staging identification. J Vis Exp.

[23] Paur H, Wright PT, Sikkel MB (2012). High levels of circulating epinephrine trigger apical cardiodepression in a beta2-adrenergic receptor/Gi-dependent manner a new model of Takotsubo cardiomyopathy. Circulation.

[24] Hou H, Zhao Z, Ong ‘achwa Machuki J (2018). Estrogen deficiency compromised the β 2 AR-Gs/Gi coupling implications for arrhythmia and cardiac injury. Pflugers Arch.

[25] Hill Brent J.F., Dalton Robin J., Joseph Biny K., Thakali Keshari M., Rusch Nancy J. (2017). 17β -estradiol reduces Cav 1.2 channel abundance and attenuates Ca2+ -dependent contractions in coronary arteries. Pharmacology Research & Perspectives.

[26] Schwertz DW, Beck JM, Kowalski JM, Ross JD (2004). Sex differences in the response of rat heart ventricle to calcium. Biol Res Nurs.

[27] Vizgirda VM, Wahler GM, Sondgeroth KL, Ziolo MT, Schwertz DW (2002). Mechanisms of sex differences in rat cardiac myocyte response to beta-adrenergic stimulation. Am J Physiol Heart Circ Physiol.

[28] Schwertz DW, Vizgirda V, Solaro RJ, Piano MR, Ryjewski C (1999). Sexual dimorphism in rat left atrial function and response to adrenergic stimulation. Mol Cell Biochem.

[29] Bergmann P, Militzer K, Schmidt P, Buttner D (1995). Sex differences in age development of a mouse inbred strain body composition, adipocyte size and organ weights of liver, heart and muscles. Lab Anim.

[30] Shutt RH, Howlett SE (2008). Hypothermia increases the gain of excitation-contraction coupling in Guinea pig ventricular myocytes. Am J Physiol Cell Physiol.

[31] Parks RJ, Ray G, Bienvenu LA, Rose RA, Howlett SE (2014). Sex differences in SR ca (2+) release in murine ventricular myocytes are regulated by the cAMP/PKA pathway. J Mol Cell Cardiol.

[32] Sims C, Reisenweber S, Viswanathan PC, Choi B-R, Walker WH, Salama G (2008). Sex, age, and regional differences in L-type calcium current are important determinants of arrhythmia phenotype in rabbit hearts with drug-induced long QT type 2. Circ Res.

[33] Yang H-Y, Firth JM, Francis AJ, Alvarez-Laviada A, MacLeod KT (2017). Effect of ovariectomy on intracellular ca (2+) regulation in Guinea pig cardiomyocytes. Am J Physiol Heart Circ Physiol.

[34] James AF, Arberry LA, Hancox JC (2004). Gender-related differences in ventricular myocyte repolarization in the Guinea pig. Basic Res Cardiol.

[35] Xiao L, Zhang L, Han W, Wang Z, Nattel S (2006). Sex-based transmural differences in cardiac repolarization and ionic-current properties in canine left ventricles. Am J Physiol Heart Circ Physiol.

[36] Mason SA, MacLeod KT (2009). Cardiac action potential duration and calcium regulation in males and females. Biochem Biophys Res Commun.

[37] Brouillette J, Lupien M-A, St-Michel C, Fiset C (2007). Characterization of ventricular repolarization in male and female Guinea pigs. J Mol Cell Cardiol.

[38] Grandy SA, Howlett SE (2006). Cardiac excitation-contraction coupling is altered in myocytes from aged male mice but not in cells from aged female mice. Am J Physiol Heart Circ Physiol.

[39] Leblanc N, Chartier D, Gosselin H, Rouleau JL (1998). Age and gender differences in excitation-contraction coupling of the rat ventricle. J Physiol.

[40] Fares Elias, Pyle W. Glen, Ray Gibanananda, Rose Robert A., Denovan-Wright Eileen M., Chen Robert P., Howlett Susan E. (2013). The Impact of Ovariectomy on Calcium Homeostasis and Myofilament Calcium Sensitivity in the Aging Mouse Heart. PLoS ONE.

[41] Papp R, Bett GCL, Lis A (2017). Genomic upregulation of cardiac Cav1.2alpha and NCX1 by estrogen in women. Biol Sex Differ.

[42] Chu SH, Sutherland K, Beck J, Kowalski J, Goldspink P, Schwertz D (2005). Sex differences in expression of calcium-handling proteins and beta-adrenergic receptors in rat heart ventricle. Life Sci.

[43] Yang Xiaoyan, Mao Xiaofang, Xu Gao, Xing Shasha, Chattopadhyay Ansuman, Jin Si, Salama Guy (2018). Estradiol up-regulates L-type Ca 2+ channels via membrane-bound estrogen receptor/phosphoinositide-3-kinase/Akt/cAMP response element-binding protein signaling pathway. Heart Rhythm.

[44] Parks RJ, Bogachev O, Mackasey M, Ray G, Rose RA, Howlett SE (2017). The impact of ovariectomy on cardiac excitation-contraction coupling is mediated through cAMP/PKA-dependent mechanisms. J Mol Cell Cardiol.

[45] Pavlaki Nikoleta, Nikolaev Viacheslav (2018). Imaging of PDE2- and PDE3-Mediated cGMP-to-cAMP Cross-Talk in Cardiomyocytes. Journal of Cardiovascular Development and Disease.

[46] Chen W, Wang R, Chen B (2014). The ryanodine receptor store-sensing gate controls Ca2+ waves and Ca^2+^-triggered arrhythmias. Nat Med.

[47] Bhupathy P, Babu GJ, Ito M, Periasamy M (2009). Threonine-5 at the N-terminus can modulate sarcolipin function in cardiac myocytes. J Mol Cell Cardiol.

[48] Schmitt JP, Ahmad F, Lorenz K (2009). Alterations of phospholamban function can exhibit cardiotoxic effects independent of excessive sarcoplasmic reticulum Ca^2+^-ATPase inhibition. Circulation..

[49] De Backer O, Debonnaire P, Gevaert S, Missault L, Gheeraert P, Muyldermans L (2014). Prevalence, associated factors and management implications of left ventricular outflow tract obstruction in takotsubo cardiomyopathy a two-year, two-center experience. BMC Cardiovasc Disord.

[50] Kaumann A, Bartel S, Molenaar P (1999). Activation of beta2-adrenergic receptors hastens relaxation and mediates phosphorylation of phospholamban, troponin I, and C-protein in ventricular myocardium from patients with terminal heart failure. Circulation.

[51] Kang S, Liu Y, Sun D, Zhou C, Liu A (2012). Chronic activation of the G protein-coupled receptor 30 with agonist G-1 attenuates heart failure. PLoS One.

[52] Xu C, Liu A, Sun H (2010). β_2_-adrenoceptor confers cardioprotection against hypoxia in isolated ventricular myocytes and the effects depend on estrogenic environment. J Recept Signal Transduct.

[53] Curl CL, Wendt IR, Kotsanas G (2001). Effects of gender on intracellular calcium in rat cardiac myocytes. Pflugers Arch.

